# Global Expression Patterns of Three *Festuca* Species Exposed to Different Doses of Glyphosate Using the Affymetrix GeneChip Wheat Genome Array

**DOI:** 10.1155/2009/505701

**Published:** 2010-02-23

**Authors:** Ozge Cebeci, Hikmet Budak

**Affiliations:** Biological Sciences & Bioengineering Program, Faculty of Engineering and Natural Sciences, Sabanci University, Orhanli, Tuzla, Istanbul 34956, Turkey

## Abstract

Glyphosate has been shown to act as an inhibitor of an aromatic amino acid biosynthetic pathway, while other pathways that may be affected by glyphosate are not known. Cross species hybridizations can provide a tool for elucidating biological pathways conserved among organisms. Comparative genome analyses have indicated a high level of colinearity among grass species and Festuca, on which we focus here, and showed rearrangements common to the Pooideae family. Based on sequence conservation among grass species, we selected the Affymetrix GeneChip Wheat Genome Array as a tool for the analysis of expression profiles of three Festuca (fescue) species with distinctly different tolerances to varying levels of glyphosate. Differences in transcript expression were recorded upon foliar glyphosate application at 1.58 mM and 6.32 mM, representing 5% and 20%, respectively, of the recommended rate. Differences highlighted categories of general metabolic processes, such as photosynthesis, protein synthesis, stress responses, and a larger number of transcripts responded to 20% glyphosate application. Differential expression of genes encoding proteins involved in the shikimic acid pathway could not be identified by cross hybridization. Microarray data were confirmed by RT-PCR and qRT-PCR analyses. This is the first report to analyze the potential of cross species hybridization in Fescue species and the data and analyses will help extend our knowledge on the cellular processes affected by glyphosate.

## 1. Introduction

Glyphosate (N-phosphonomethylglycine) is a broad spectrum herbicide that affects plants systemically after application to the leaf surface. It is phytotoxic and prevents further growth by blocking aromatic amino acid production, leading to the arrest of protein synthesis and secondary compound formation. It specifically inhibits 5-enolpyruvylshikimate-3-phosphate synthase (EPSPS), a nuclear encoded chloroplast-localized enzyme in the shikimic acid pathway of plants and microorganisms [1]. 

Although it is relatively inexpensive and less toxic to nontarget organisms, glyphosate has not been extensively used in turfgrass weed management programs due to its possible adverse effects on turfgrass growth [2]. Until now, glyphosate usage has been limited to spot treatments. However, in the presence of natural glyphosate-tolerant turfgrass species, such as cool-season perennial turfgrass, there is an increased reliance on the usage of glyphosate for weed control [2]. The development of cultivars with greater tolerance to glyphosate is considered to be a good alternative for weed control using this environmentally friendly herbicide in lawns, golf courses, and other turf areas. Additionally, determining the effective glyphosate rate that can be used directly on turfgrass fields to control weeds is essential for extensive usage of this herbicide. Evolution of resistance to other herbicides, with different modes of action, increased reliance on the herbicide glyphosate for weed control [3]. A better understanding of its action on turfgrass species is essential for the development of future management strategies both to slow down the evolution of resistance and to control existing populations [4]. 

Microarray hybridization is a valuable tool to analyze whole genome expression changes upon any treatment. However, a commercial array platform is not available for turfgrass species. Cross-Species Hybridization (CSH) is a new and useful tool to perform a large-scale functional profiling without an available genome sequence to identify genes that are conserved among species throughout evolution. In addition, it offers an important tool for identifying molecular mechanisms and pathways conserved among species [5–7]. These studies included CSH analysis of highly diverged species, *Caenorhabditis elegans* and *Drosophila melanogaster* [5], and of more related organisms, *Candida albicans* and *Saccharomyces cerevisiae* [7]. In a recent study [8], mechanisms controlling embryonic stem cell (ESC) pluripotency were investigated by comparing gene expression patterns of human and mouse ESC orthologous genes. Another recent study reported that usage of a multispecies cDNA array identified conserved genes expressed in oocytes. Gene sequences from three organisms, bovine, mouse, and *Xenopus laevis,* diverged in their evolutionary position, have been utilized to design a multispecies cDNA array for the identification of conserved sequences playing roles in molecular mechanisms or pathways common to all species [9]. In both studies evolutionarily distant species were selected to identify common mechanisms and pathways. Additionally, a comparison of results obtained by CSH using species specific hybridization (SSH) proved that biological processes analyzed by CSH closely reflected the analysis found by SSH [10].

The Affymetrix GeneChip Wheat Genome Array was selected to identify global gene expression changes in three selected fescues. The rationale for selecting the wheat genome array for the CSH experiment was based on the close relatedness of perennial ryegrass, which is relatively similar to fescues, to the Triticeae [11]. In the same study, the existence of synteny and colinearity among the genetic maps of ryegrass and Triticeae cereals has been postulated. Triticeae, ryegrass, and fescues reside in the same subfamily, Pooideae of the Poaceae family [12]. High level of similarity in terms of gene order among these families makes it feasible to consider CSH to reveal the cross-species conservation of biological processes and their genetic control mechanisms. Festuca species were selected for their differential glyphosate tolerance based on dry matter production, chlorophyll content, and shoot concentration of shikimic acid [4]. Based on these morphological and physiological data, selected genotypes were used to analyze and understand global expression changes upon glyphosate treatments. Large-scale functional profiling of Festuca species with differential tolerance to glyphosate treatment will be a beneficial resource for future investigations concerning biochemical effects of glyphosate on turfgrasses.

## 2. Materials and Methods

### 2.1. Plant Materials

Seeds of three different turfgrass species, Ambrose (*Festuca rubra *subsp. *falax*), Cindy Lou (*Festuca rubra* subsp. *littoralis*), and Discovery (*Festuca brevipila*) were directly planted on soil and grown under controlled conditions in the greenhouse with daytime and nighttime temperatures of 25°C and 20°C, respectively. Glyphosate [RoundUp Ultra; acid equivalent (a.e.): 356 g  L^−1^ N-[phosphonomethyl]glycine, Monsanto Co.] treatment was performed four weeks after sowing by spraying a total volume of 100 mL of either 5% or 20% solution (1.58 mM and 6.32 mM, resp.) directly on the leaves under open air conditions. Plants at the three-leaf growth stage were sprayed with freshly prepared glyphosate solution until all leaves were fully wet (about 10 mL) but without run-off. Control plants were sprayed with distilled water. Leaf samples were collected 5 days after treatment.

### 2.2. RNA Isolation

Total RNA isolations were carried out by Trizol reagent (Invitrogen) according to the manufacturer's instructions. Three separate RNA isolations were performed for each glyphosate dose of each species. RNA concentrations were determined spectrophotometrically and RNA qualities were checked by denaturing gel before the microarray analysis.

### 2.3. Gene Chip Analysis

All hybridizations were performed as biological triplicates of control and glyphosate treated samples (RNA isolation and cRNA labeling were done separately for each hybridization) of three Festuca genotypes and two glyphosate doses according to the manufacturer's instructions. Cell intensity files were analyzed using Partek Genomics Suite version 6.3 Beta (Partek incorporated) with robust multichip average normalization [13]. The data quality was confirmed by PCA (Principal Component Analysis) and box-whisker plots. Analysis of variance (ANOVA) was used to further analyze the log-transformed expression with the defined threshold expression values of *P* < 0.1 and DE < − 2 or DE > 2. Raw data is deposited in to the ArrayExpress database (http://www.ebi.ac.uk/). Cluster and Treeview programs were used for identification of differentially expressed probes [14, 15].

### 2.4. RT-PCR Analysis

RT-PCR analysis was performed as outlined by Cebeci et al. [16] with minor modifications. cDNAs were quantified spectrophotometrically and diluted to 400 ng *μ*L^−1^. One *μ*L of this cDNA was amplified with 0.5 *μ*M of gene specific primers and 18S rRNA primers in a total of 20 *μ*L volume. The primer pair selected for RT validation was specific to alternative oxidase because it was found to be up-regulated in all three species. Primers for Germin-Like protein 1 precursor, Chlorophyll a/b-binding protein WCAB precursor, and Thylakoid membrane phosphoprotein 14 kDa, chloroplast precursor were also designed for RT validation. Sequences of the primers designed to amplify (1) a region of 196 bp are as follows: TA.233.1.S1_AT (Alternative oxidize), forward: CGTCCACTCCTACACCGAGT/reverse: TGGTAGTACACGTCCGATGC; (2) a region of 156 bp, TA.28351.1.S1_AT (Germin-like protein 1 precursor) forward: GGCCTGCAGATCACTGACTA, reverse: CACGACGAACTTTGCTGAGA; (3) a region of 150 bp, TA.30702.1.S1_X_AT (Chlorophyll a/b-binding protein WCAB precursor) forward: GGAGATCAAGAACGGTCGTC, reverse: ACGAAGTTAGTGGCGAATGC. (4) a region of 207 bp, TA.636.1.S1_S_AT Thylakoid membrane phosphoprotein 14 kDa, chloroplast precursor forward: ACGAAGTTAGTGGCGAATGC, reverse: TTCCAGGTCTCATGGAGGTC. PCR amplification was performed with 18S rRNA primers (forward: ATGATAACTCGACGGATCGC/reverse: CTTGGATGTGGTAGCCGTTT) and actin (GenBank accession AY663392, forward: GGATCTCACGGACTCCCTCAT/reverse: CGGCTGAGGTTGTGAAGGA) as control reaction. 

### 2.5. Quantitative Real-Time RT-PCR (qRT-PCR)

Three micrograms of total RNA were used for first strand cDNA synthesis using the Superscript III reverse transcriptase (Invitrogen), quantified spectrophotometrically and diluted to 400 ng *μ*L^−1^. One *μ*L of this cDNA was amplified with 0.8 *μ*M of specific primers in a total of 20 *μ*l volume using SYBR green PCR master mix (Applied Biosystems) with Icycler Multicolor Real-time PCR Detection Systems (Bio-Rad Laboratories) [17, 18]. The quantification was performed using actin (GenBank accession AY663392, forward: GGATCTCACGGACTCCCTCAT/reverse: CGGCTGAGGTTGTGAAGGA) as an internal reference and three independent PCR results with acceptable efficiency (1.8–2.2) were averaged. Quantitative real-time RT-PCR analyses were performed for four probes selected for RT-PCR analysis. 

## 3. Results and Discussion

### 3.1. Cross-Species Hybridization Analysis

 Gene expression profiles of three Festuca species, Ambrose, Cindy Lou, and Discovery, were examined in response to increasing levels of glyphosate using the Affymetrix GeneChip Wheat Genome Array. This array contains 61,127 probe sets representing *Triticum aestivum, T. turgidum*, *T. turgidum *ssp. *durum*, *T. monococcum*, and *Aegilops tauschii *transcripts. The CSH approach led to significant differential regulation of only 1337 probe sets (231 probes from Ambrose, 767 from Cindy Lou, and 339 probes from Discovery) at the defined threshold expression values of *P* < .1 and DE < − 2 or DE > 2. Although only 2.2% of the probes in total stayed above the threshold, biologically meaningful information could be extracted from this data set, which could be used to elucidate conserved mechanisms responsive to glyphosate common to fescues and wheat. The low percentage of hybridization might be explained by the presence of interspecies differences between the probe and target sequences. The single nucleotide polymorphisms may result in alteration of probe-hybridization affinities and hence, generate lower hybridization signal intensities [19]. Much higher hybridization ratios have been reported in recent studies exploiting CSH with cDNA arrays [8, 9] because cDNA platforms are likely more suitable for CSH studies owing to the longer cDNA probes. The wheat array platform chosen in our study appears to provide a benefit in that it enabled the detection and identification of highly conserved genes common to fescues and wheat, such as photosynthesis or reactive oxygen species scavenging.

### 3.2. Gene Expression Profiles in Festuca Species

**Figure 1:**
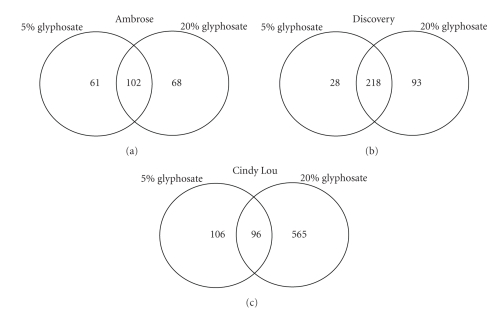
Venn diagrams representing the total number of differentially regulated probes in three Festuca genotypes in response to two glyphosate doses, 5% and 20%. (a) Ambrose; (b) Discovery; (c) Cindy Lou.

The number of differentially expressed probes increased proportionally at 20% foliar glyphosate treatment. Hybridization with the Cindy Lou led to detection of a larger number of probes (Figure 1(c)), whereas Ambrose showed the lowest number of differentially expressed probes (Figure 1(a)). The total number of differentially expressed probe sets in Discovery was intermediate to that of Ambrose and Cindy Lou in response to glyphosate (Figure 1(b)). Interestingly, the number of probes with differential expression was almost constant in cultivar Ambrose irrespective of the glyphosate application, whereas the number of probes increased with 20% glyphosate application for both Discovery and Cindy Lou with a strong response in the latter (Figure 1). 

**Figure 2:**
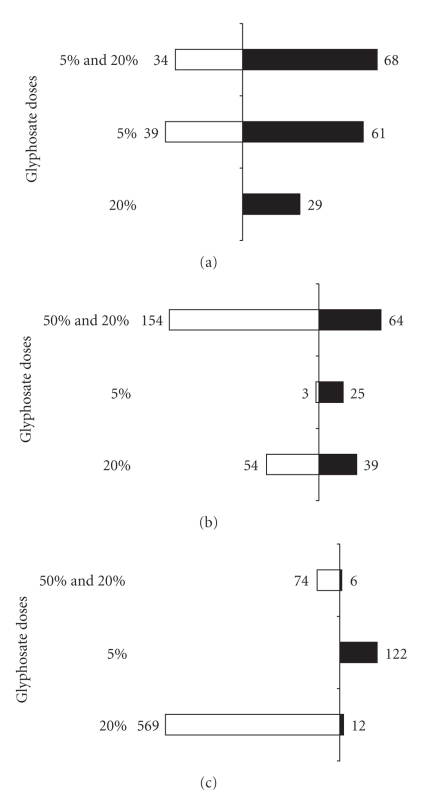
The total number of differentially regulated probe sets (*P* < .1) in glyphosate applied plants. (a) Ambrose; (b) Discovery; (c) Cindy Lou. Up-regulated probes are represented by black bars, whereas down-regulated probes are represented by white bars.

In Cindy Lou, most of the differentially expressed probes were found to be up-regulated at the 5% glyphosate application dose, but this pattern was opposite for the 20% glyphosate application (Figure 2(c)). Transcripts altered with 20% glyphosate applications were mostly down-regulated. In contrast, for Ambrose, the number of up-regulated probes was more than the down-regulated probes for both glyphosate doses (Figure 2(a)). 

**Figure 3:**
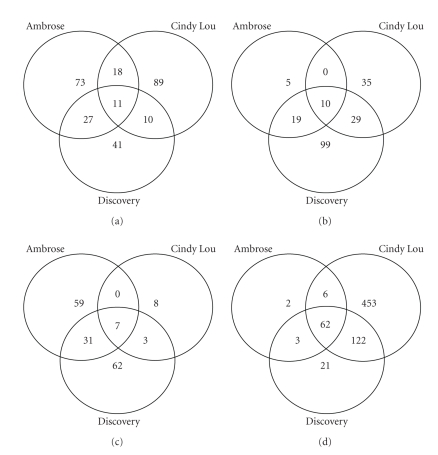
Venn diagrams representing the total number of differentially regulated probe sets common to three Festuca species. (a) Up-regulated by 5% glyphosate application. (b) Down-regulated by 5% glyphosate application. (c) Up-regulated by 20% glyphosate application. (d) Down-regulated by 20% glyphosate application.

The total number of common up-regulated probes in Ambrose and Discovery at both glyphosate doses was greater than the number of common probes in Discovery and Cindy Lou (Figure 3). These results were opposite for down-regulated probes. The mechanisms controlled by common down-regulated probe sets are likely more conserved among Cindy Lou and Discovery and appear to initiate a response to the higher doses of glyphosate.

### 3.3. Functional Analysis

**Figure 4:**
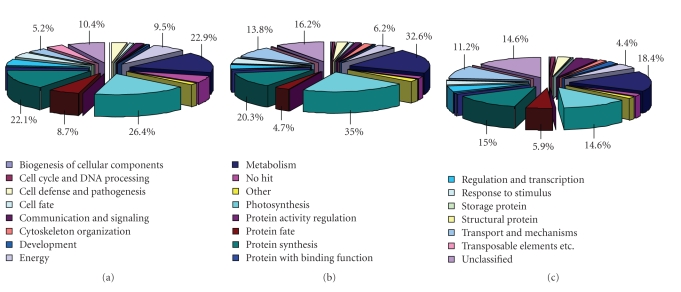
Representation of the functional annotation of all differentially regulated probes in Festuca species. (a) Ambrose, (b) Discovery, (c) Cindy Lou.

**Figure 5:**
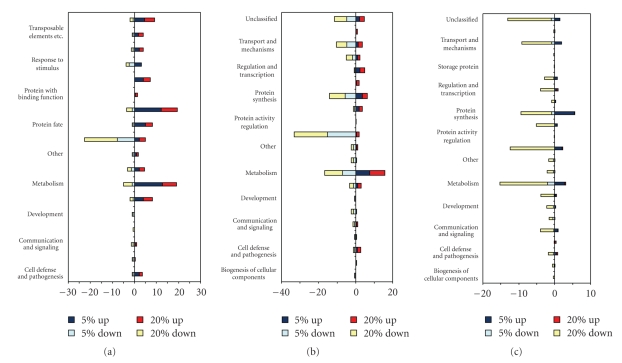
Functional categories of largest differentially regulated probe sets in three Festuca genotypes. (a) Ambrose, (b) Discovery, (c) Cindy Lou exposed to 5% and 20% glyphosate.

Probes with differential expression were annotated by homology searches of target sequences using BLASTn in the TIGR wheat and rice genome databases (http://www.tigr.org/) and the GenBank nr database (http://www.ncbi.nlm.nih.gov/). Subsequently, we searched for functions using the ExPASy proteomics server [20] (Figure 4). Differentially expressed probes were grouped into 21 functional categories according to MIPS functional categories (http://www.mips.gsf.de/). The largest probe sets were categorized under “Photosynthesis” (~25.3%, average of three genotypes), “Metabolism” (~24.6%), “Protein Synthesis” (~19.1%), “Unclassified” (~13.7%), “Transport & Mechanisms” (~10.1%), “Energy” (~6.7%), and “Protein Fate” (~6.4%) (Figure 4). As could be expected, probes in the group “Photosynthesis” were down-regulated in all genotypes since one of the secondary responses of plants to glyphosate is the inhibition of photosynthesis via several routes [21–24]. In Ambrose, all differentially expressed probes except the ones in the photosynthesis category were found to be up-regulated at both glyphosate doses (Figure 5(a)), suggesting that most biological processes, except photosynthesis, were active in this genotype or showed enhanced expression. As for Cindy Lou, the transcript abundance pattern was found to be different from Ambrose. A major portion of the probes residing in the listed categories were up-regulated by the 5% glyphosate application. However, increasing glyphosate treatment to 20% resulted in down regulation of most probes (Figure 5(c)). These results support our proposal that glyphosate leads to the induction of a more profound down-regulatory response when it is applied at a relatively higher dose (20%) in Cindy Lou. This result was expected based on a previous report indicating that glyphosate led to the inhibition of many biological processes, including chlorophyll synthesis, plant tissue ion fluxes, and activity of anti-oxidative enzymes [25]. As for Discovery, most probes played roles in protein synthesis, photosynthesis, and transport mechanisms which were down-regulated (Figure 5(b)). Inhibition of protein synthesis was an expected response, because the major mode of action of glyphosate is inhibition of aromatic amino acid biosynthesis, and hence protein synthesis [26]. 

**Figure 6:**
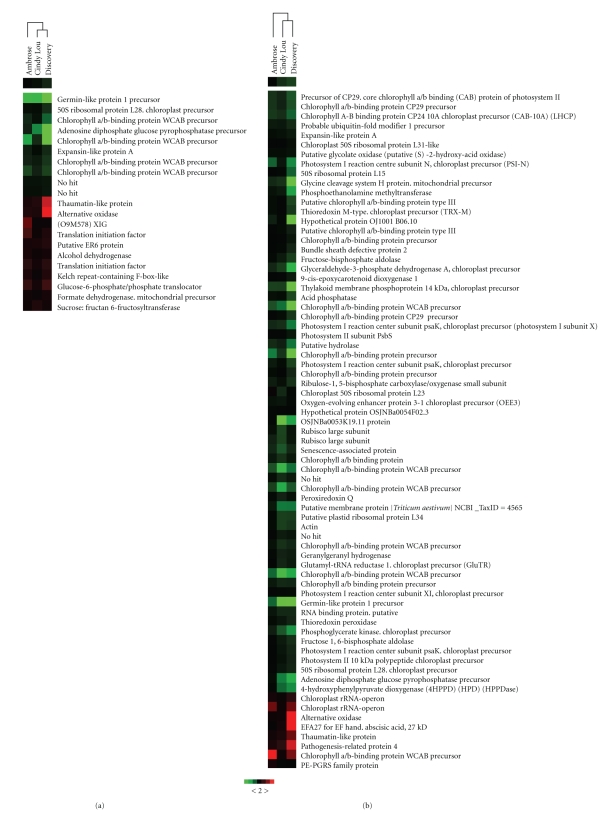
Cluster analysis of probes with differential regulation in three Festuca genotypes. (a) 5% glyphosate (b) 20% glyphosate. The color saturation reflects the fold change where green is for more than 2 fold down-regulated and red is for more than 2 fold up-regulated probes with *P* < .1.

Cluster analysis using differentially expressed probes common to all genotypes for both glyphosate rates showed that differentially expressed probes in all Festuca genotypes in response to 5% (21 probes) and 20% glyphosate (71 probes) treatment grouped separately. The Treeview results indicated that differentially expressed probes of Cindy Lou and Discovery clustered together for both glyphosate rates (Figures 6(a) and 6(b)). Ambrose was shown to cluster separately from Cindy Lou and Discovery, which are proposed to be more tolerant to glyphosate in comparison to Ambrose [4]. In other words, transcriptome changes in Cindy Lou and Discovery at both glyphosate rates resembled each other. 

### 3.4. Reverse Transcriptase (RT) and Quantitative Real-Time (qRT) PCR Analyses

**Figure 7:**
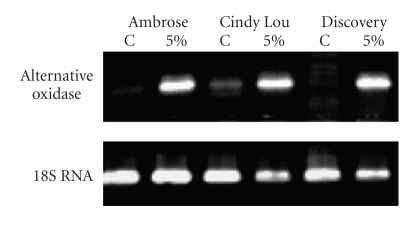
A representative picture of validation of microarray result using RT PCR for one common Festuca probe set.

RT-PCR was performed on both control and three 5% glyphosate-treated Festuca to validate our CSH results. The candidate probe, TA.233.1.S1_AT, was found to have homology to “Alternative Oxidase” by functional analysis and was differentially expressed in all three genotypes. Sequencing of the amplified product in Fescue showed a high level of similarity wheat alternative oxidase genes (*Triticum aestivum*, *E* value 1e−90). This probe was up-regulated at the 5% glyphosate treatment, in accordance with the CSH data (Figure 7). Amplification and functional analysis of “Thylakoid membrane phosphoprotein 14 kDa”, “Germin-Like protein 1 precursor”, and “Chlorophyll a/b-binding protein WCAB precursor” confirmed our CSH results. QRT-PCR performed on selected probes (Alternative oxidase, Germin-like protein 1 precursor, Chlorophyll a/b-binding protein WCAB precursor, chloroplast precursor) showed that microarray and qRT-PCR results were in good agreement (*r* = 93) with respect to trends of regulation. 

### 3.5. Glyphosate Treatment Led to Down-Regulation of Photosynthesis Related Genes

Analysis of the differentially expressed probes with roles in photosynthesis revealed that glyphosate led to the down-regulation of most probes related to photosynthesis in all Festuca species at both glyphosate doses. This reduction in gene expression was mostly apparent in transcripts functioning in chlorophyll biosynthesis, photosystem activities, and RuBisCo, a key player in the Calvin cycle (Table 1). The decline in transcript abundance was more pronounced for plants treated with the higher glyphosate dose.

A major mode of action by glyphosate affects the aminolevulinic acid (ALA) pathway or the porphyrin biosynthesis pathway. In the ALA pathway, glyphosate interferes with the activity of aminolevulinate synthase preventing the conversion of succinyl CoA (from the tricarboxylic acid cycle) to ALA. Blockage of this step in porphyrin biosynthesis leads to a decline of compounds containing porphyrin, such as chlorophyll [27, 28]. Additionally, it has been reported that leaf chlorophyll content of plants exposed to sublethal doses of glyphosate is lower [21, 22]. Hence, one of the primary reasons for the decline in expression levels of probes playing a role in photosynthesis might be related to the deleterious effect of glyphosate on chlorophyll. Glyphosate treatment was also shown to inhibit photosynthesis by blocking the allocation of carbon to starch [23], and resulted in an immediate and rapid decline in the level of ribulose bisphosphate and associated photosynthetic carbon metabolism in sugar beet [24]. These studies are consistent with the reduction in the levels of transcripts related to photosynthetic pathways being linked to the inhibitory effect of glyphosate in Festuca species. 

### 3.6. Regulation of Detoxification of Reactive Oxygen Species (ROS) is Genotype Dependent

ROS generation causes oxidative damage to membrane lipids, DNA, and proteins [29]. CSH-based transcript profiling of Festuca species indicated that glyphosate treatment at different rates leads to the down-regulation of transcripts involved in the detoxification of ROS. The major strategy used by plants to tolerate oxidative stress is the production of anti-oxidative enzymes that convert ROS to less toxic compounds. In a previous study, it has been shown that glyphosate will exert its deleterious effects on maize plants by amplifying lipid peroxidation of biomembranes (MDA) [25]. Additionally, it was postulated that the appearance of a small number of changes in lipid peroxidation and antioxidative defense mechanisms in susceptible and resistant soybean cultivars exposed to sublethal doses of glyphosate [30] In this study, probes with homology to antioxidative enzymes, such as putative glutathione peroxidase, thioredoxin peroxidase, and peroxiredoxin Q were down-regulated. The major reason for down-regulation of these peroxidases might be the inhibition of ALA by glyphosate action.

This is the first report to analyze the potential of cross-species hybridization in Fescue species and the data and analyses will help extend our knowledge on the cellular processes affected by glyphosate. This study is important for paving the way to better understand the mechanisms and pathways regulating glyphosate responses of Festuca species. 

## Figures and Tables

**Table 1a:** (a) List of common or species-specific probes related to (a) photosynthesis, (b) oxidative stress, and (c) cell signaling and differentially expressed in response to two different doses of glyphosate. Given are the fold changes calculated by ANOVA. Up-regulation fold changes are given in bold letters, whereas down-regulation by italics. No significant differential expression is shown with an empty cell. Ambrose probe fold changes are underlined, Discovery are double-underlined, and Cindy Lou are nonunderlined.

		**Ambrose**		**Discovery**		**Cindy Lou**	
		**Fold Change**					
		**5%**	**20%**	**5%**	**20%**	**5%**	**20%**

**Probe Set ID**	**Target Sequence**						

**Ta.27761.1.S1_x_at**	Photosystem I reaction center subunit psaK, chloroplast precursor	*−3,30122*	*−13,2487*	*−3,01112*	*−37,0513*	*—*	*−6,62621*
**Ta.27751.3.S1_x_at**	Photosystem I reaction center subunit XI, chloroplast precursor	*—*	*−3,53086*	*−3,03125*	*−6,11773*	*—*	*−5,63676*
**Ta.28750.1.S1_at**	Photosystem II 10 kDa polypeptide chloroplast precursor	*—*	*−2,6278*	*−4,16983*	*−17,2639*	*—*	*−8,04824*
**Ta.1161.1.S1_at**	Photosystem II subunit PsbS	*—*	*−3,47497*	*—*	*−11,5118*	*—*	*−3,58749*
**Ta.1139.1.S1_at**	Precursor of CP29, core chlorophyll a/b binding (CAB) protein of photosystem II	*−5,04791*	*−28,6882*	*−7,37361*	*−46,5839*	*—*	*−18,6875*
**Ta.28265.1.S1_at**	Oxygen-evolving enhancer protein 3-1, chloroplast precursor (OEE3)	*—*	*−8,69806*	*−3,77085*	*−5,07437*	*—*	*−10,209*
**Ta.30702.1.S1_x_at**	Chlorophyll a/b-binding protein WCAB precursor	*−40,9861*	*−58,6499*	*−64,5336*	*−98,1407*	*−10,1345*	*−119,736*
**Ta.20639.3.S1_x_at**	Chlorophyll a/b-binding protein precursor	*−3,23933*	*−6,25985*	*−6,19484*	*−18,7793*	*—*	*−2,68378*
**TaAffx.128414.219.S1_x_at**	Rubisco large subunit	*−9,75494*	*−16,4849*	*−3,08632*	*−10,8861*	*—*	*−37,5039*
**Ta.2752.2.S1_x_at**	Ribulose-1,5-bisphosphate carboxylase/oxygenase small subunit	*—*	*−14,2542*	*—*	*−27,3256*	*—*	*−7,39744*

**Table 1b:** (b)

		**Cindy Lou**		**Discovery**	
		**Fold Change**			
**Probe Set ID**	**Target Sequence**	**5%**	**20%**	**5%**	**20%**

Ta.28714.1.S1_at	Thioredoxin peroxidase	*—*	*−10,1624*	*−2,57569*	*−17,9772*
Ta.6572.1.S1_a_at	Peroxiredoxin Q	*—*	*−17,3425*	*−4,85985*	*−7,80475*
Ta.18063.2.S1_at	Putative glutathione peroxidase	*−2,69247*	*−5,38618*	*—*	*—*
Ta.547.1.S1_at	Cytosolic glutathione reductase	*—*	*—*	**9,12843**	**11,6039**
Ta.14644.2.S1_x_at	Superoxide dismutase [Cu-Zn] 4A	*—*	*—*	**2,52839**	**2,62177**

**Table 1c:** (c)

		**Cindy Lou**		**Discovery**	
		**Fold Change**			
**Probe Set ID**	**Target Sequence**	**5%**	**20%**	**5%**	**20%**

Ta.6269.1.S1_at	Putative serine/threonine kinase 38	*—*	*−3,59976*		*—*
Ta.991.1.S1_a_at	Serine/threonine-protein kinase SAPK8	*—*	*−7,70463*	*—*	*—*
TaAffx.86456.1.S1_s_at	Putative calcium/calmodulin-dependent protein kinase CaMK	*—*	*−6,76413*	*—*	*—*
Ta.11837.1.S1_at	Calmodulin	*—*	*−5,03177*	*—*	*—*
Ta.6979.1.S1_s_at	Phosphatidylinositol 3-and 4-kinase family-like,	*—*	*−40,9189*	*—*	*—*
Ta.1890.1.S1_x_at	Nt-iaa28 deduced protein	*−2,85957*	*−2,85957*	*—*	*—*
Ta.6968.2.S1_a_at	Ethylene-responsive small GTP-binding protein	*—*	*−2,60726*	*—*	*—*
Ta.25390.1.S1_s_at	(Q91W51) WASP family 1	*—*	*—*	**3,49126**	**4,82506**
